# Ferulic Acid Exerts Neuroprotective Effects via Autophagy Induction in *C. elegans* and Cellular Models of Parkinson's Disease

**DOI:** 10.1155/2022/3723567

**Published:** 2022-02-22

**Authors:** Tao Long, Qian Wu, Jing Wei, Yong Tang, Yan-Ni He, Chang-Long He, Xue Chen, Lu Yu, Chong-Lin Yu, Betty Yuen-Kwan Law, Jian-Ming Wu, Da-Lian Qin, An-Guo Wu, Xiao-Gang Zhou

**Affiliations:** ^1^Sichuan Key Medical Laboratory of New Drug Discovery and Drugability Evaluation, Luzhou Key Laboratory of Activity Screening and Druggability Evaluation for Chinese Materia Medica, Key Laboratory of Medical Electrophysiology of Ministry of Education, School of Pharmacy, Southwest Medical University, Luzhou, Sichuan 646000, China; ^2^Central Nervous System Drug Key Laboratory of Sichuan Province, Luzhou, Sichuan 646000, China; ^3^Department of Ophthalmology in the Affiliated Hospital of Southwest Medical University, Luzhou, Sichuan 646000, China; ^4^State Key Laboratory of Quality Research in Chinese Medicine, Macau University of Science and Technology, Macau 999078, China

## Abstract

Parkinson's disease (PD) is a complex neurological disorder characterized by motor and nonmotor features. Although some drugs have been developed for the therapy of PD in a clinical setting, they only alleviate the clinical symptoms and have yet to show a cure. In this study, by employing the *C. elegans* model of PD, we found that ferulic acid (FA) significantly inhibited *α*-synuclein accumulation and improved dyskinesia in NL5901 worms. Meanwhile, FA remarkably decreased the degeneration of dopaminergic (DA) neurons, improved the food-sensing behavior, and reduced the level of reactive oxygen species (ROS) in 6-OHDA-induced BZ555 worms. The mechanistic study discovered that FA could activate autophagy in *C. elegans*, while the knockdown of 3 key autophagy-related genes significantly revoked the neuroprotective effects of FA in *α*-synuclein- and 6-OHDA-induced *C. elegans* models of PD, demonstrating that FA exerts an anti-PD effect via autophagy induction in *C. elegans*. Furthermore, we found that FA could reduce 6-OHDA- or H_2_O_2_-induced cell death and apoptosis in PC-12 cells. Moreover, FA was able to induce autophagy in stable GFP-RFP-LC3 U87 cells and PC-12 cells, while bafilomycin A1 (Baf, an autophagy inhibitor) partly eliminated the protective effects of FA against 6-OHDA- and H_2_O_2_-induced cell death and ROS production in PC-12 cells, further confirming that FA exerts an anti-PD effect via autophagy induction in vitro. Collectively, our study provides novel insights for FA as a potent autophagy enhancer to effectively prevent neurodegenerative diseases such as PD in the future.

## 1. Introduction

Parkinson's disease (PD) is the second most common neurodegenerative disorder clinically characterized by the aggregation of *α*-synuclein and the progressive loss of dopaminergic (DA) neurons in the substantia nigra [1]. DA neurodegeneration causes primary motor symptoms, including tremors, bradykinesia, and muscle rigidity, and eventually personality, behavioral, and cognitive symptoms, including anxiety, depression, dementia, and sleep disruption [1]. Although the cause of PD remains to be defined fully, it most likely results from the complex interactions between a variety of genetic and environmental risk factors, such as mutations in specific genes and neurotoxin exposure [2]. For example, *α*-synuclein protein is encoded by the SNCA gene (PARK1) and is abundant in the presynaptic terminals of neurons in the brain. However, there is evidence showing that cytoplasmic Lewy bodies composed of the abnormal accumulation of mutant *α*-synuclein are closely associated with sporadic and familial PD [2]. In addition, 6-hydroxydopamine (6-OHDA), the oxidative metabolite of dopamine, is toxic and is detected in the brain and urine of PD patients [3]. Massive researches have revealed that 6-OHDA selectively enters DA neurons via the dopamine active transporter (DAT) and then promotes the overproduction of ROS and causes irreversible damage to DA neurons, ultimately leading to the degeneration or death of DA neurons in the brain of PD patients [4]. Currently, there are no effective treatments for PD, and the existing medications, including levodopa (L-Dopa), dopamine agonists (pramipexole and ropinirole), monoamine oxidase B inhibitors (selegiline and rasagiline), and coenzyme Q10, can only alleviate the symptoms of PD [5]. Therefore, the discovery of novel drugs is an urgent demand for the treatment of PD, and targeting the inhibition of *α*-synuclein and the decrease in damage induced by PD toxins may be promising strategies for the treatment of PD.

Autophagy is an evolutionarily conserved pathway in which damaged or superfluous cell components are delivered by autophagosomes to lysosomes for degradation and then recycled back into the cytosol [6]. The growing evidence has demonstrated that autophagy plays a protective mechanism in neurodegenerative diseases by timely degrading toxic/aggregated proteins and damaged organelles [7, 8]. The free-living nematode *Caenorhabditis elegans* (*C. elegans*) is an ideal model that has been widely used in the study of neurodegenerative diseases such as PD [9]. In addition, there are numerous autophagy-related genes, including *lgg-1*, *sqst-1*, *unc-51*, *vps-34*, and *atg-18* (homologous to human *LC3B*, *P62*, *Ulk-1*, *Vps-34*, and *Atg-18*, respectively), which have been originally identified in yeast and subsequently identified in nematodes, mice, and mammals [10, 11]. In this study, the N5901 strain expressing *α*-synuclein-GFP and the BZ555 strain [Pdat-1::gfp] were used as the *C. elegans* models of PD. The BC12921 strain expressing *sqst-1*-GFP and the DA2123 strain expressing *lgg-1*-GFP were employed to evaluate the autophagy effect of ferulic acid (FA) in nematodes. Moreover, 6-OHDA, a PD neurotoxin, and H_2_O_2_ were used to induce neuronal damage in PC-12 cells, and the autophagy effect was determined in stable GFP-RFP-LC3 U87 cells and PC-12 cells.

In recent years, natural products such as bioactive compounds or extracts derived from traditional Chinese medicines (TCMs) have received great attention worldwide and shown bright prospects in the treatment of neurodegenerative diseases, including PD [12]. FA belonging to hydroxycinnamic acid is naturally found in a variety of TCMs, such as *Rhizoma Ligustici* wallichii, *Angelica sinensis*, and *Asafoetida giantfennel* [13]. Several studies have revealed that FA possesses multiple pharmacological activities, including antioxidative, anti-inflammatory, antimicrobial, antiviral, and anticancer effects [14]. However, the neuroprotective effects and molecular mechanisms of FA in PD are still unclear. In present study, the neuroprotective effects of FA were investigated in transgenic *C. elegans* strain including NL5901 and BZ555 worms, as well as in 6-OHDA- and H_2_O_2_-induced PC-12 cells. In addition, the autophagic effect of FA was determined in BC12921 and DA2123 worms and stable GFP-RFP-LC3 U87 and PC-12 cells. Finally, we found that FA inhibited *α*-synuclein accumulation, improved behavioral ability, and decreased the degeneration of DA neurons via autophagy induction in vivo and in vitro.

## 2. Materials and Methods

### 2.1. Chemicals, *C. elegans* Strains, Culture, and Synchronization

Ferulic acid (FA, F103701), bafilomycin A1 (Baf, B101389), and levodopa (L-Dopa, D111048) were purchased from Aladdin Bio-Chem Technology Co., Ltd. (Shanghai, China). 6-Hydroxydopamine (6-OHDA, H4381), 5-fluoro-2′-deoxyuridine (FUDR, F0503), and rapamycin (Rap, V900930) were purchased from Sigma-Aldrich (St. Louis, USA). *C. elegans* strains used in this study were as follows: wild-type Bristol N2, transgenic BZ555 strain [Pdat-1::gfp], N5901 strain [unc-54p::alpha synuclein::YFP + unc-119(+)], BC12921 strain [rCes T12G3.1::GFP + pCeh361], and DA2123 strain [lgg-1p::GFP::lgg-1 + rol-6(su1006)]. All strains were obtained from the Caenorhabditis Genetics Center (CGC) and cultured at 20°C on nematode growth medium (NGM) agar plates carrying a lawn of *Escherichia coli* OP50 as a food source unless otherwise stated. Synchronized eggs (embryos) were isolated from gravid adults by bleaching solution (0.5 M NaOH and 1% NaClO) and incubated in M9 buffer at 20°C overnight to obtain synchronized L1 larvae. Then, L4 larval worms were transferred onto NGM plates containing 5 mg/L FUDR to prevent progeny from hatching.

### 2.2. Locomotion Assay

Age-synchronized NL5901 nematodes were bred on NGM agar plates with or without FA to reach the L4 developmental stage. Then, worms were transferred onto new NGM plates containing FUDR (5 mg/L) to prevent the growth of progeny. When the animals grew to L4 + 5 or L4 + 10 days, they were independently placed in a drop of M9 buffer and allowed to recover for 90 s to avoid observing behavior caused by stress. Then, the number of body bends was counted in 20 s. At least 20 animals were used to analyze in each experiment, and all experiments were carried out in triplicate.

### 2.3. Quantitative Analysis of *α*-Synuclein Accumulation

NL5901 nematodes were used to determine the accumulation of *α*-synuclein protein as reported previously [15, 16]. In brief, synchronized NL5901 L1 larvae were incubated on NGM plates with or without FA (50 *μ*M) for 7 days and 12 days at 20°C. Then, worms were washed with M9 buffer 3 times and transferred to 2% agarose pad slides with 100 mM sodium azide. Immobilized animals were captured under a positive fluorescence microscope (Leica DM6B, Leica Microsystems GmbH, Germany) to monitor the accumulation of *α*-synuclein, and the fluorescence intensity was quantified by using ImageJ software (National Institutes of Health, Bethesda, MD, USA). At least 20 animals were used to analyze in each group, and all experiments were carried out in triplicate.

### 2.4. Quantitative Analysis of DA Neurodegeneration

The assay of DA neurodegeneration was performed in 6-OHDA-induced BZ555 worms as described previously [15, 16]. In brief, synchronized L3 BZ555 worms were incubated with 50 mM 6-OHDA and 10 mM ascorbic acid in S-medium blended with OP50 in the presence or absence of L-Dopa or FA. After treatment for 1 h, the worms were washed with M9 buffer 3 times and transferred onto NMG plates containing L-Dopa or FA for 24 h. Then, 5 mg/L of FUDR was added to inhibit the production of progeny. After 48 h of treatment, BZ555 worms were washed 3 times with M9 buffer and then mounted onto a glass slide with 2% agarose pad using 100 mM sodium azide and enclosed with a coverslip. The immobilized animals were observed and photographed under a positive fluorescence microscope, and the fluorescence intensity of DA neurons was measured by using ImageJ software. At least 20 animals were used to analyze in each group, and all experiments were carried out in triplicate.

### 2.5. Food-Sensing Behavioral Test

The food-sensing behavioral test was performed in 6-OHDA-induced BZ555 worms according to the method described previously [15, 16]. In brief, synchronized L3 BZ555 worms were incubated with 50 mM 6-OHDA and 10 mM ascorbic acid in S-medium blended with OP50 in the presence or absence of L-Dopa or FA for 1 h. Then, worms were washed with M9 buffer 3 times and transferred onto NMG plates containing L-Dopa or FA. After 72 h, BZ555 worms were transferred to the center of NGM plates spotted with or without *E. coli* OP50 lawn and allowed to recover for 90 s to avoid observing behavior caused by stress. Then, the numbers of body bending of worms were counted in 20 s intervals. The slowing rate of body bending was calculated by the following formula:

(1) $$\begin{array}{c} \text{Slowing rate}=\frac{N_{\text{without food}}-N_{\text{with food}}}{N_{\text{without food}}}, \end{array}$$

where *N* represents the total number of body bending in the presence or absence of bacteria.

### 2.6. Measurement of ROS Levels in *C. elegans*

For H_2_O_2_-induced N2 worms, 4 gravid adults were allowed to lay eggs for 6 h in NGM plates containing H_2_O_2_ (1 mM) in the presence or absence of FA (50 *μ*M) or NAC (5 mM). Then, the adult worms were picked out, and the NGM plates were continued to culture at 20°C for 72 h. After treatment, worms were washed with M9 buffer and incubated in 1 mL M9 containing 100 *μ*M dihydroethidium (DHE) for 1 h in the dark. After incubation, the tubes were centrifuged at a speed of 2000 rpm, and the worms were mounted onto a glass slide in M9 medium containing 100 mM sodium azide. Images were captured using a positive fluorescence microscope. For quantifying the RFP fluorescence intensity, images were analyzed and quantified by ImageJ software.

### 2.7. Survival Assay of *C. elegans*

The survival assay of *C. elegans* under acute oxidative stress was performed according to the previous study [17]. Briefly, synchronized L1 N2 worms were maintained at 20°C and treated with FA for 48 h. After that, worms were transferred to new NGM medium containing 40 mM H_2_O_2_, and the survival rate of worms was counted after 0-12 h of H_2_O_2_ treatment.

### 2.8. Measurement of Autophagy Effect in *C. elegans*

The autophagy effect of FA was monitored by counting GFP-positive LGG-1/Atg8 puncta in the hypodermal seam cells of DA2123 strain and by detecting the GFP fluorescence of p62/SQST-1-GFP fusion protein in the BC12921 strain. In brief, the two strains were maintained at 20°C and treated with FA or Rap (a positive control) on the day after eggs hatching for 48 h. Then, the worms were mounted onto a glass slide with 2% agarose pad using 100 mM sodium azide to observe and capture representative photographs under a positive fluorescence microscope. The fluorescence intensity representing the total number of GFP::LGG-1/Atg8 puncta in the DA2123 strain or indicating p62 expression in the BC12921 strain was measured by using ImageJ software as previously described [18].

### 2.9. RNA Interference

RNA interference (RNAi) of the 3 key autophagy-related genes including *lgg-1*, *vps-34*, and *unc-51* was performed according to the previously described procedures [19]. In brief, the synchronized L1 worms of NL5901 or BZ555 were transferred onto the NGM plates with the *E. coli* HT115 bacteria expressing the double-stranded RNA of individual genes or the control bacteria HT115 in the presence or absence of FA. After treatment, the adult nematodes were used for the analysis of *α*-synuclein and ROS accumulation and degeneration of DA neurons as described above.

### 2.10. Cell Culture and Treatment

Stable GFP-RFP-LC3 U87 cells were a generous gift kindly provided by Dr. Xiaoming Zhu (Macau University of Science and Technology, Macao, China) and cultured in *α*-MEM supplemented with 10% fetal bovine serum (FBS) and 50 U/mL penicillin and 50 *μ*g/mL streptomycin (Invitrogen, Scotland, UK). PC-12 cells were purchased from the American Type Culture Collection (ATCC) (Rockville, MD, USA). PC-12 cells were cultured in DMEM supplemented with 10% horse serum (HS), 5% FBS, and 50 U/mL penicillin and 50 *μ*g/mL streptomycin. All cells were maintained in the 5% CO_2_ incubator with 75% humidity at 37°C. To prevent direct interaction between FA and 6-OHDA and H_2_O_2_ in the culture medium, PC-12 cells were pretreated with FA for 12 h prior to the treatment of 6-OHDA or H_2_O_2_. At the end of FA treatment, the medium was exchanged for fresh medium containing 6-OHDA or H_2_O_2_. Then, the PC-12 cells were incubated with 6-OHDA or H_2_O_2_ for another 12 h.

### 2.11. MTT Assay

The PC-12 cells were seeded onto 96-well plates at a density of 4 × 10^3^ cells/well and treated with the test drugs for 24 h. After treatment, 10 *μ*L of MTT solution (5 mg/mL) was added into each well followed by incubation for 4 h. Then, the medium was removed, and 100 *μ*L of DMSO was added into each well. After shaking at a low speed for 10 min at room temperature, the colorimetric reading of each well was obtained with a spectrophotometer (BioTek, VT Lab, USA) at 570 nm. The cytotoxicity was measured by calculating the cell viability according to the following formula: cell viability (%) = 100 × OD value_Cells treated_/OD value_Cells untreated_. The data were obtained from three separate experiments.

### 2.12. Flow Cytometry Analysis

Cell apoptosis was measured with a flow cytometer using the Annexin V-FITC/PI Detection Kit (BD Biosciences, CA, USA). In brief, after treatment, cells were collected and centrifuged at a speed of 2000 rpm for 5 min. The supernatant was removed, and the cell pellet was resuspended with 500 *μ*L of 1× Annexin V solution containing 4 *μ*L of propidium iodide (PI) and 2 *μ*L of FITC solution according to the manufacturer's instructions. After 15 min incubation in the dark, the cells were analyzed with a FACSVerse flow cytometer (BD Biosciences, San Jose, CA, USA). Data acquisition and analysis were performed by using BD FACSuite v1.0.6 software (BD Biosciences, San Jose, CA, USA).

### 2.13. Hoechst/PI Staining Assay

PC-12 cells were seeded onto 96-well plates at a density of 4 × 10^3^ cells/well and treated with the test drugs for 24 h. After treatment, PC-12 cells were washed with PBS 3 times. After that, the cells were stained with 5 mg/L Hoechst 33342 and 5 mg/L PI solution for 5 min. After incubation, the liquid was sucked dry, and 100 *u*L of PBS was added into wells. Representative images were captured and analyzed under a fluorescence microscope (Nikon ECLIPSE 80i, Tokyo, Japan). The cell apoptosis was measured by calculating the percentage of cells with PI signal (red fluorescence) in cells with Hoechst signal (blue fluorescence).

### 2.14. Measurement of ROS Levels in PC-12 Cells

The intracellular ROS levels were determined as previously described by flow cytometry analysis using the H_2_DCFDA fluorescence probe [20]. Briefly, after treatment, PC-12 cells were trypsinized and collected for centrifugation at a speed of 1000 rpm for 5 min. Then, the cell pellet was washed twice with PBS. After that, the supernatant was removed, and the remaining cell pellet was then resuspended in PBS with 20 *μ*M H_2_DCFDA and continued to incubate for 20 min in the dark. After incubation, the cell suspension was then subjected to flow cytometry analysis for the measurement of ROS levels on a FACSVerse flow cytometer (BD Biosciences, San Jose, CA, USA). Data acquisition and analysis were performed by using the FlowJo software (BD Biosciences, San Jose, CA, USA).

### 2.15. Quantification of GFP-LC3 Puncta Formation in Cells

For the quantification of GFP-LC3 puncta formation, stable GFP-RFP-LC3 U87 cells were employed. In brief, cells were treated with FA at the indicated concentrations or Rap (0.5 *μ*M) for 24 h. After treatment, the cells were fixed with 4% paraformaldehyde (PFA) for 20 min at room temperature, followed by the wash with PBS twice. Then, the slides were brought out for air-drying and mounted by using FluorSave^™^ mounting media (Calbiochem, San Diego, CA, USA). Representative images of cells were captured by using a Nikon ECLIPSE 80i fluorescence microscope equipped with a CCD digital camera Spot RT3^™^ (Diagnostic Instruments, Inc., Melville, NY, USA). The autophagic effect was evaluated by calculating the total number of punctate GFP-LC3 fluorescence formations in the GFP-positive cells. In this study, at least 60 GFP-positive cells were counted from 3 randomly selected fields of the slides.

### 2.16. Statistical Analysis

All data in this study were obtained from three or more independent experiments. The data expressed as means ± SD were analyzed by using the GraphPad Prism 6.0 software (San Diego, CA, USA) using one-way ANOVA followed by the pos hoc Tukey test. *p* < 0.05 was considered to have significant differences among the compared groups.

## 3. Results

### 3.1. FA Reduces *α*-Synuclein Protein Levels and Improves Motility in NL5901 *C. elegans*

**Figure 1:**
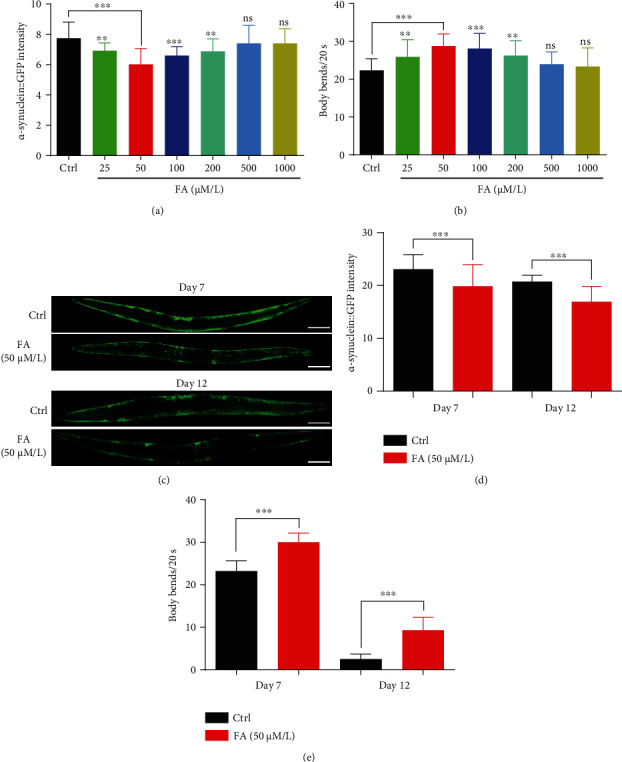
FA inhibits the accumulation of *α*-synuclein and enhances the body locomotion in NL5901 worms. (a) The bar chart indicates the fluorescence intensity representing the *α*-synuclein protein levels of NL5901 worms after 7 days of FA treatment at the indicated concentrations. (b) The body bends of NL5901 worms were counted after the treatment of FA at the indicated concentrations for 7 days. The bar chart indicates the number of body bends in 20 s. (c) Representative fluorescence images of NL5901 worms treated with FA (50 *μ*M) were captured by using a fluorescence microscope on day 7 and day 12. Scale bars: 100 *μ*m. (d) The bar chart indicates the fluorescence intensity representing the *α*-synuclein protein levels of NL5901 worms treated with FA (50 *μ*M) for 7 and 12 days. (e) The bar chart indicates the body bends of NL5901 worms treated with FA (50 *μ*M) for 7 and 12 days. Data were collected from at least 20 worms and expressed as the mean ± SD. ^ns^*p* > 0.05, ^∗∗^*p* ≤ 0.01, and ^∗∗∗^*p* ≤ 0.001.

In this study, we investigated the effect of FA on the inhibition of *α*-synuclein accumulation and the improvement of motility in transgenic NL5901 nematodes. It is reported that NL5901 worms express human *α*-synuclein fused with yellow fluorescent protein (YFP) under the control of the *unc-54* promoter in the body wall muscle cells [21], and there are three advantages in this model. First, the *unc-54* promoter is powerful, and muscle cells are the largest and easiest cell type to score, which allows accurate detection of *α*-synuclein accumulation and its subcellular localization. Second, the expression and subsequent aggregation of *α*-synuclein in the muscle result in PD-like progressive decline of motility in *C. elegans*, demonstrating the in vivo toxicity of *α*-synuclein aggregates [22]. Third, this model has been successfully used to identify compounds possessing anti-PD effects and modifier genes of PD in previous reports [23, 24]. In the present study, we found that 25–200 *μ*M of FA significantly decreased the YFP intensity in NL5901 worms (Figure 1(a)), suggesting that FA can inhibit the accumulation of *α*-synuclein in vivo. Meanwhile, 25–200 *μ*M of FA also improved the motor ability of NL5901 worms, as evidenced by the increased number of body bends in 20 s (Figure 1(b)). Among these concentrations, 50 *μ*M of FA exhibited the best effect. Thus, 50 *μ*M was selected for FA as the optimal concentration in the subsequent experiments. In addition, we further investigated the inhibitory effect of FA on the accumulation of *α*-synuclein in NL5901 worms at different adulthood stages. The results showed that FA treatment significantly reduced the YFP intensity of NL5901 worms at both day 7 (day 5 of adults) and day 12 (day 10 of adults) with the reduced rates of 13.9% and 18.3%, respectively (Figures 1(c) and 1(d)). With the production and aggregation of *α*-synuclein, the locomotor capacity of NL5901 worms decreases with aging. It was found that FA significantly alleviated the motor dysfunction of NL5901 worms (Figure 1(e)). Taken together, our results demonstrate that FA can ameliorate the pathology of PD in *C. elegans*, manifesting in the reduction of *α*-synuclein accumulation and the improvement of locomotor capacity.

### 3.2. FA Decreases the Degeneration of DA Neurons in 6-OHDA-Induced BZ555 *C. elegans*

**Figure 2:**
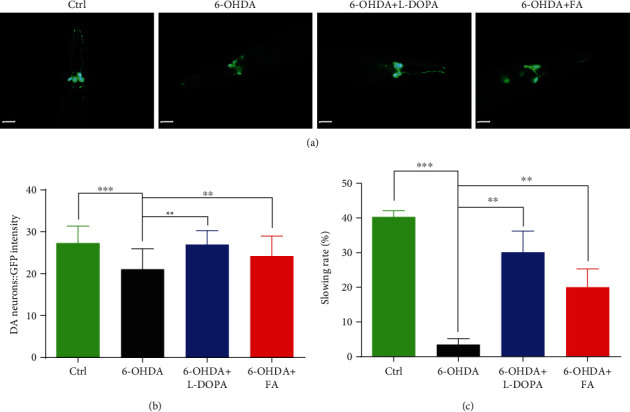
FA inhibits DA neurodegeneration induced by 6-OHDA in BZ555 worms. (a) Representative images of DA neurons with GFP signals were captured in BZ555 worms treated with FA (50 *μ*M) or L-Dopa (2 mM) in the presence or absence of 6-OHDA (50 mM) for 72 h. Scale bars: 20 *μ*m. (b) The bar chart indicates the GFP intensity representing the content of DA neurons in BZ555 worms, which was quantified by using the ImageJ software. (c) The bar chart indicates the relative slowing rate (%) of BZ555 worms treated with FA (50 *μ*M) or L-Dopa (2 mM) in the presence or absence of 6-OHDA (50 mM) for 72 h. Data were collected from at least 20 worms and expressed as the mean ± SD. ^∗∗^*p* ≤ 0.01 and ^∗∗∗^*p* ≤ 0.001.

There is a growing body of evidence indicating that the degeneration or death of DA neurons is one of the main pathological features of PD [1]. *C. elegans* possess exactly eight DA neurons, containing two pairs of cephalic (CEP) neurons and one pair of anterior deirid (ADE) neurons in the head region and one pair of posterior deirid (PDE) neurons in the posterior lateral region [23, 25]. It has been reported that 6-OHDA can enter into these DA neurons through the DAT-1 dopamine transporter and thus trigger their degeneration in worms, while the addition of L-Dopa, which serves as the precursor of dopamine and is converted to dopamine by aromatic l-amino acid decarboxylase, leads to an increase in dopamine levels, thus effectively protecting DA neuron loss in 6-OHDA-exposed worms [26]. In this study, we evaluated whether FA protected against the degeneration of DA neurons using the transgenic BZ555 nematodes. It specifically expressed the green fluorescent protein (GFP) in DA neurons under the promoter of the *dat-1* gene (P*dat-1*::GFP), which encodes for the dopamine transporter, DAT-1. Due to the specific fluorescent expression in DA neurons, P*dat-1*::GFP was used as the reporter construct to monitor the changes of dopaminergic neuronal viability [27]. As expected, 6-OHDA exposure led to the degeneration of DA neurons in BZ555 worms, as indicated by the decreased GFP intensity. However, the supplement of FA or L-Dopa remarkably recovered the GFP intensity to 15.0% and 27.8%, respectively (Figures 2(a) and 2(b)). These results suggest that FA and L-Dopa inhibit 6-OHDA-induced DA neurodegeneration in *C. elegans*.

**Figure 3:**
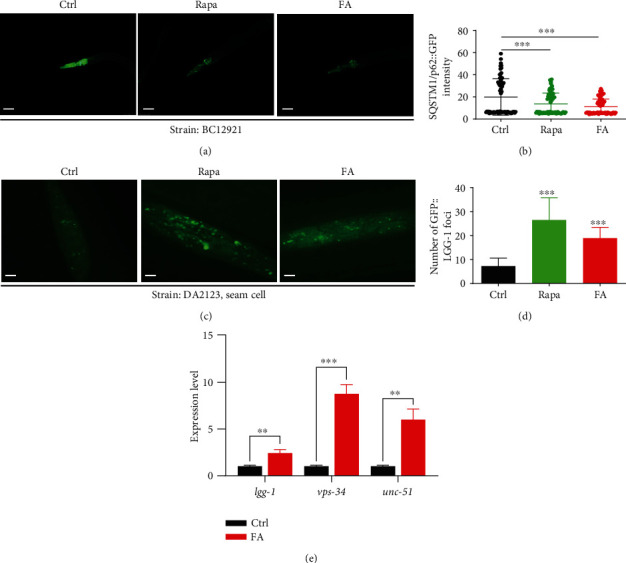
FA induces autophagy in *C. elegans*. (a) BC12921 worms with the GFP signal were treated with FA (50 *μ*M) or Rap (20 *μ*M) for 48 h. Then, the representative images of worms were captured by using a fluorescence microscope. Scale bars: 50 *μ*m. (b) The bar chart indicates the GFP intensity reflecting the expression of SQSTM1/p62 proteins in BC12921 worms. Data were collected from at least 20 worms and expressed as the mean ± SD. ^∗∗∗^*p* ≤ 0.001. (c) Representative images of DA2123 worms treated with FA (50 *μ*M) or Rap (20 *μ*M) for 48 h were captured by using a fluorescence microscope. Scale bars: 20 *μ*m. (d) The bar chart indicates the mean number of GFP::LGG-1 puncta in the same size area of DA2123 worms. Data were collected from at least 20 worms and expressed as the mean ± SD. ^∗∗∗^*p* ≤ 0.001. (e) N2 worms were treated with FA for 48 h. Then, qRT-PCR was performed to analyze the mRNA expression of 3 autophagy-related genes. The bar chart indicates the relative expression levels of *lgg-1*, *vps-34*, and *unc-51*. Data were collected from three independent experiments and expressed as the mean ± SE. ^∗∗^*p* ≤ 0.01 and ^∗∗∗^*p* ≤ 0.001.

In addition, it has been confirmed that 6-OHDA-induced BZ555 worms exhibit the food-sensing behavioral deficit [28]. Normally, *C. elegans* bend their bodies for transportation and foraging. When *C. elegans* come across the bacteria lawn or food source, they decline their bending frequency to feed themselves more effectively. Thus, the bending frequency is commonly used to estimate the food-sensing behavior of worms. As shown in Figure 2(c), the BZ555 worms without treatment exhibited a decline in bending frequency with a slowing rate of 40.5% upon contact with the bacteria food. However, 6-OHDA-induced BZ555 worms failed to decrease the bending frequency with a slowing rate of 3.6%. After treatment with FA or L-Dopa, 6-OHDA-induced BZ555 worms markedly slowed down their bending frequency, and their slowing rates were improved to 20.1% and 30.3%, respectively (Figure 3(c)). Collectively, these results indicate that FA decreases the degeneration of DA neurons in 6-OHDA-induced BZ555 *C. elegans*.

### 3.3. FA Reduces the Generation of Intracellular ROS in 6-OHDA-Induced BZ555 *C. elegans*

**Figure 4:**
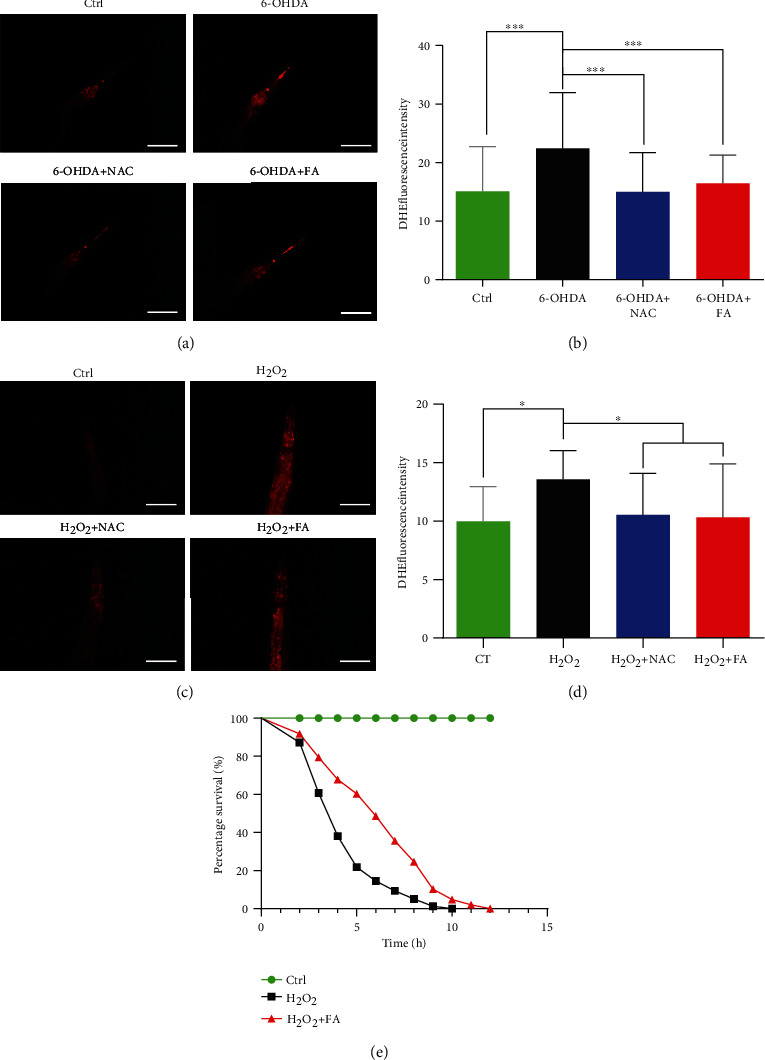
FA reduces the ROS levels in 6-OHDA- or H_2_O_2_-induced BZ555 or N2 worms. (a) BZ555 worms treated with FA (50 *μ*M) or L-Dopa (2 mM) for 72 h in the presence or absence of 6-OHDA (50 mM) were incubated with DHE solution (100 *μ*M) for 1 h. Then, the representative images of worms with RFP signals were captured by using a fluorescence microscope. Scale bars: 20 *μ*m. (b) The bar chart indicates the relative fluorescence intensity of DHE, which represents the ROS levels of BZ555 worms treated with 6-OHDA. Data were collected from at least 20 worms and were expressed as the mean ± SD. ^∗∗^*p* ≤ 0.01. (c) N2 worms treated with FA (50 *μ*M) or NAC (5 mM) for 72 h in the presence or absence of H_2_O_2_ (1 mM) were incubated with DHE solution (100 *μ*M) for 1 h. Then, the representative images of worms with RFP signals were captured by using a fluorescence microscope. Scale bars: 20 *μ*m. (d) The bar chart indicates the relative fluorescence intensity of DHE, which represents the ROS levels of N2 worms. Data were collected from at least 20 worms and expressed as the mean ± SD. ^∗∗∗^*p* ≤ 0.001. (e) The survival rate of N2 worms treated with FA (50 *μ*M) in the presence or absence of H_2_O_2_ (40 mM) was determined at different points. Data were collected from at least 60 worms for each treatment.

Previous studies have proved that FA is an effective scavenger of free radicals, suggesting that it possesses great antioxidant capacity [29]. Here, we investigated whether FA could eliminate the excessive ROS generation in 6-OHDA-induced BZ555 worms using the fluorescent dihydroethidium (DHE) probe. As shown in Figures 4(a) and 4(b), the BZ555 worms in the 6-OHDA group exhibited a significant increase in DHE intensity compared to the untreated BZ555 worms, while the treatment with FA or L-Dopa could attenuate the DHE intensity in 6-OHDA-induced BZ555 worms. Therefore, these data suggest that FA inhibits the generation of ROS and exerts antioxidative activity in 6-OHDA-induced BZ555 worms.

### 3.4. FA Decreases ROS Levels and Extends Lifespan in H_2_O_2_-Treated N2 *C. elegans*

To further confirm the antioxidative effect of FA in *C. elegans*, H_2_O_2_ was used to induce oxidative stress in wild-type N2 nematodes, and the ROS levels were measured using the fluorescent DHE probe. The results showed that H_2_O_2_-treated worms exhibited an increase in the DHE fluorescence signal, which was significantly reduced by the supplement of FA or NAC, suggesting that FA can reduce the ROS levels in H_2_O_2_-treated *C. elegans* (Figures 4(c) and 4(d)). In addition, we also assessed the resistance of FA to acute oxidative stress induced by 40 mM H_2_O_2_. By measuring the lifespan of nematodes, we found that FA significantly increased the survival rate of *C. elegans* (Figure 4(e)), suggesting that FA extends the lifespan of H_2_O_2_-treated *C. elegans* via the antioxidation mechanism.

### 3.5. FA Induces Autophagy in *C. elegans*

Mounting evidence demonstrates that autophagy is a crucial cellular catabolic process involved in the degradation of various cytosolic components, while the defects in autophagy are critically associated with aging and aging-related diseases such as PD. In this study, two *C. elegans* strains including BC12921 and DA2123 were employed to evaluate the autophagy induction of FA in vivo. In the BC12921 strain, the autophagy substrate protein SQST-1/p62 fused to GFP is expressed under the control of the promoter of the *sqst-1* gene. In the DA2123 strain, a number of puncta labeled with GFP::LGG-1 accumulate in the hypodermal seam cells. Our results showed that the treatment with FA or Rap significantly reduced the GFP intensity in BC12921 worms, suggesting that FA or Rap can inhibit the expression of SQST-1/p62 protein (Figures 3(a) and 3(b)). Meanwhile, the treatment with FA or Rap remarkably increased the number of LGG-1-GFP-positive puncta in DA2123 worms (Figures 3(c) and 3(d)). Furthermore, qRT-PCR results showed that FA administration significantly increased the mRNA expression levels of 3 key autophagy-related genes including *lgg-1*, *vps-34*, and *unc-51* in N2 worms. Collectively, these data strongly demonstrate that FA induces autophagy in *C. elegans*.

### 3.6. FA Inhibits the Accumulation of *α*-Synuclein and Improves the Locomotor Capacity via Autophagy Induction in NL5901 *C. elegans*

**Figure 5:**
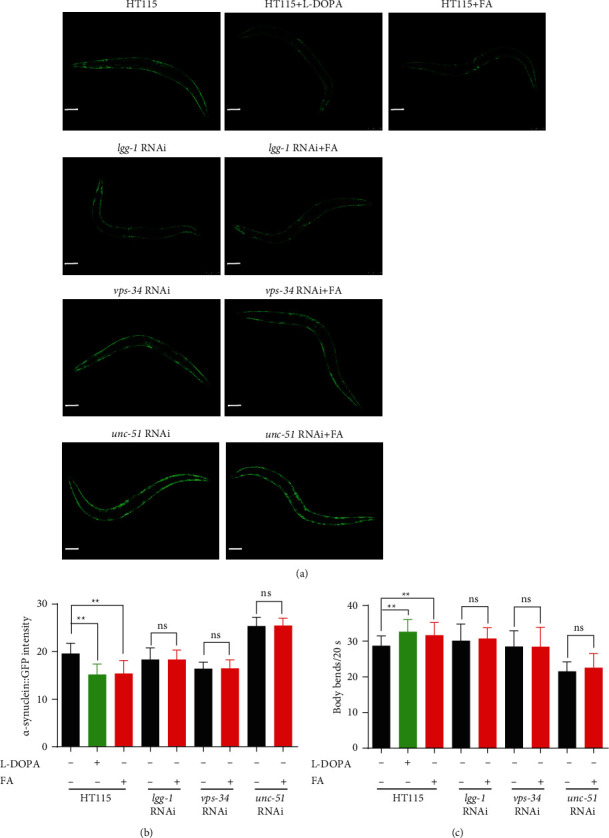
FA inhibits *α*-synuclein pathology in NL5901 worms via autophagy induction. (a) NL5901 worms fed with HT115 bacteria or RNAi bacteria of *lgg-1*, *vps-34*, or *unc-51* genes were treated with or without FA (50 *μ*M) for 7 days. Representative images with GFP signals were captured by using a fluorescence microscope. Scale bars: 100 *μ*m. (b) The bar chart indicates the relative GFP intensity representing the expression of *α*-synuclein proteins in NL5901 worms. (c) The bar chart indicates the number of body bends of NL5901 worms in 20 s. Data were collected from at least 20 worms and were expressed as the mean ± SD. ^ns^*p* > 0.05 and ^∗∗^*p* ≤ 0.01.

To investigate whether FA inhibits the accumulation of *α*-synuclein in NL5901 worms via autophagy induction, we knocked down autophagy-related genes by feeding NL5901 worms with RNAi bacteria and then examined the expression of *α*-synuclein. As shown in Figures 5(a) and 5(b), the treatment of FA or L-Dopa significantly reduced the accumulation of *α*-synuclein in NL5901 worms supplied with the control bacteria HT115. However, the effect of FA on the inhibition of *α*-synuclein was largely compromised when NL5901 worms were fed with the RNAi bacteria of 3 autophagy-related genes including *lgg-1*, *vps-34*, and *unc-51*. In addition, the mobility of NL5901 worms was also evaluated by counting the body bending frequency of worms in 20 s. The results showed that the treatment with FA or L-Dopa significantly enhanced the locomotor capacity of NL5901 worms fed with the control bacteria HT115, while the feeding of RNAi bacteria eliminated the effect of FA on the improvement of locomotion capacity (Figure 5(c)). Together, these data suggest that FA degrades *α*-synuclein and improves the locomotion capacity of NL5901 *C. elegans* via autophagy induction.

### 3.7. FA Reduces DA Neuron Degeneration and ROS Levels and Improves the Food-Sensing Behavior via Autophagy Induction in 6-OHDA-Induced BZ555 *C. elegans*

**Figure 6:**
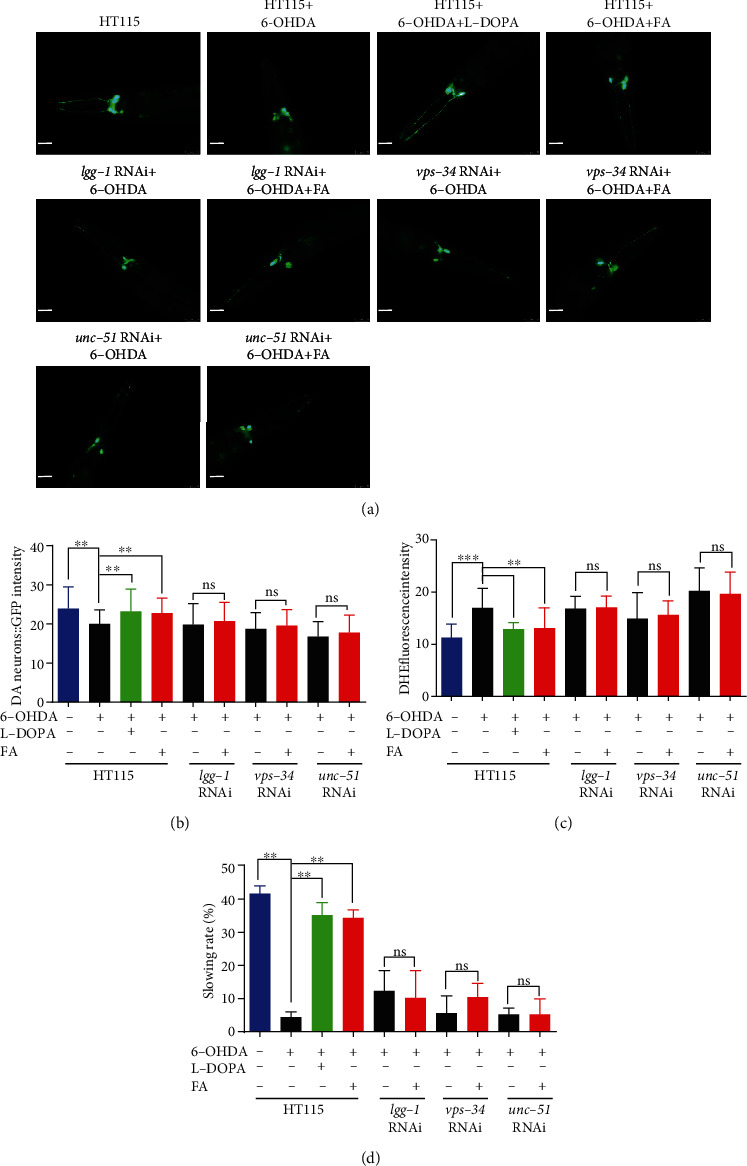
FA inhibits DA neuron degeneration and ROS levels and enhances the food-sensing behavior in 6-OHDA-induced BZ555 *C. elegans* via autophagy induction. (a) 6-OHDA-induced BZ555 worms fed with HT115 bacteria or RNAi bacteria of *lgg-1*, *vps-34*, or *unc-51* genes were treated with or without FA (50 *μ*M) or L-Dopa (2 mM) for 72 h. Representative images of BZ555 worms with GFP signals in DA neurons were captured by using a fluorescence microscope. Scale bars: 25 *μ*m. (b) The bar chart indicates the relative intensity of GFP, which represents the content of DA neuron in BZ555 worms. (c) The bar chart indicates the ROS levels of BZ555 worms after feeding with the RNAi bacteria of *lgg-1*, *vps-34*, or *unc-51* in the presence or absence of FA (50 *μ*M) or L-Dopa (2 mM). (d) The bar chart indicates the relative slowing rate (%) of BZ555 worms. Data were collected from at least 20 worms and expressed as the mean ± SD. ^ns^*p* > 0.05, ^∗∗^*p* ≤ 0.01, and ^∗∗∗^*p* ≤ 0.001.

It has been reported that autophagy dysfunction is closely related to the degeneration of DA neurons in neurodegenerative diseases [8]. In this study, we further studied whether FA inhibited the degeneration of DA neurons in 6-OHDA-induced BZ555 worms via autophagy induction. The results showed that the treatment with FA or L-Dopa significantly inhibited the degeneration of DA neurons in 6-OHDA-induced BZ555 worms, which was indicated by the recovered GFP intensity (Figures 6(a) and 6(b)). However, the feeding of RNAi bacteria targeting autophagy-related genes including *lgg-1*, *vps-34*, and *unc-51* successfully eliminated the effect of FA on the inhibition of DA neuron degeneration in 6-OHDA-induced BZ555 worms (Figures 6(a) and 6(b)). Meanwhile, the feeding of RNAi bacteria also attenuated the improvement effects of FA on the ROS accumulation and food-sensing behavior in 6-OHDA-induced BZ555 worms (Figures 6(c) and 6(d)). Taken together, these data suggest that FA inhibits the DA neuron degeneration and ROS production and improves the food-sensing behavior in 6-OHDA-induced BZ555 *C. elegans* via autophagy induction.

### 3.8. FA Improves the Cell Viability and Reduces the ROS Levels in 6-OHDA- or H_2_O_2_-Treated PC-12 Cells

**Figure 7:**
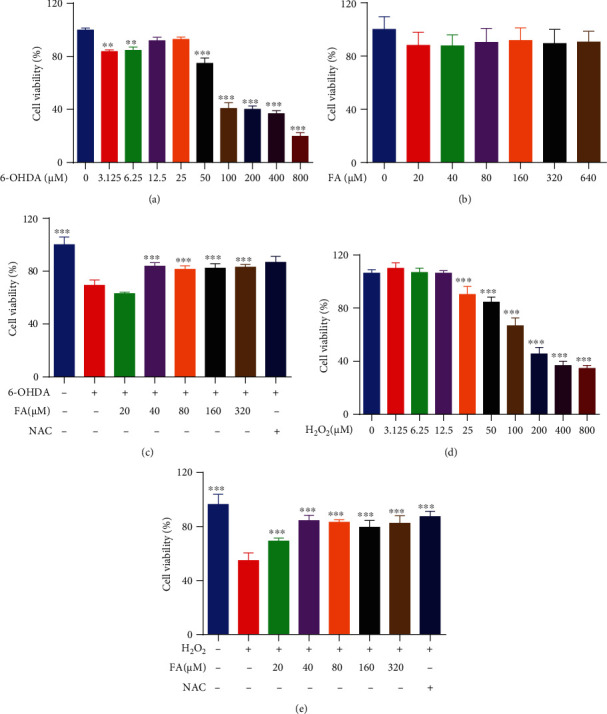
FA increases the cell viability of 6-OHDA- or H_2_O_2_-treated PC-12 cells. (a, b) Cytotoxicity of 6-OHDA or FA in PC-12 cells after 24 h of treatment was determined by the MTT assay. The IC_50_ values of 6-OHDA and FA were calculated by using GraphPad Prism 6.0 software. (c) PC-12 cells were treated with 6-OHDA in the presence or absence of FA at the indicated concentrations for 24 h. Then, the cell viability of PC-12 cells was determined by the MTT assay. The bar chart indicates the cell viability of PC-12 cells. (d) Cytotoxicity of H_2_O_2_ in PC-12 cells after 24 h of treatment was determined by the MTT assay. The IC_50_ values of H_2_O_2_ were calculated by using GraphPad Prism 6.0 software. (e) PC-12 cells were treated with H_2_O_2_ in the presence or absence of FA at the indicated concentrations for 24 h. The bar chart indicates the cell viability of PC-12 cells. Data were collected from three independent experiments and expressed as the mean ± SD. ^∗∗^*p* ≤ 0.01 and ^∗∗∗^*p* ≤ 0.001.

**Figure 8:**
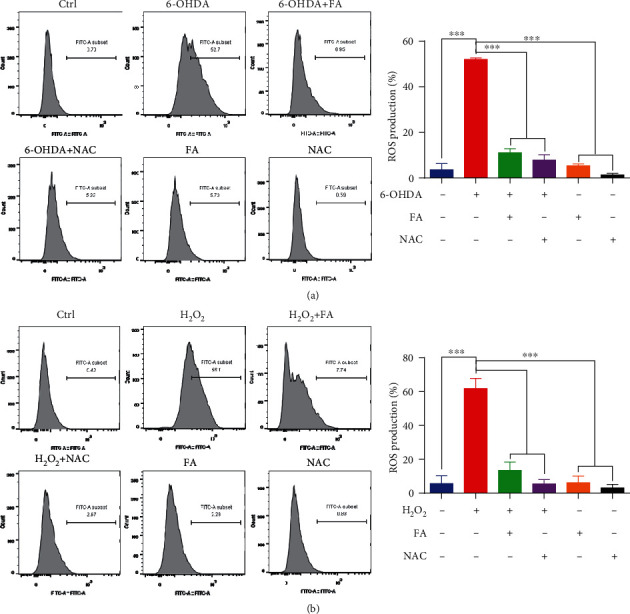
FA reduces the ROS levels in 6-OHDA- or H_2_O_2_-treated PC-12 cells. (a, b) PC-12 cells were treated with FA or NAC in the absence or presence of 6-OHDA (a) or H_2_O_2_ (b) at the indicated concentrations for 24 h. After treatment, the cells were incubated with an H_2_DCFDA probe and the GFP intensity was analyzed by flow cytometry. The bar chart presents the GFP intensity indicating the ROS levels in PC-12 cells under the indicated treatments. Data were collected from three independent experiments and expressed as the mean ± SD. ^∗∗∗^*p* ≤ 0.001.

To determine whether FA exerts the neuroprotective effects in vitro, the PD neurotoxin 6-OHDA was used to induce oxidative damage in PC-12 cells. Firstly, the cytotoxicity of 6-OHDA and FA against PC-12 cells was examined by the MTT assay (Figures 7(a) and 7(b)). Then, the cytoprotective effect of FA in 100 *μ*M of 6-OHDA-induced PC-12 cells was evaluated. The results showed that FA dose-dependently recovered the viability of PC-12 cells (Figure 7(c)). It is known that the neurotoxicity of 6-OHDA is closely associated with the generation of ROS and the possible direct inhibition of the mitochondrial respiratory chain complex I [30]. Therefore, we here investigated the cytoprotective effect of FA in the H_2_O_2_-induced oxidative damage model of PC-12 cells. The MTT assay showed that the cell viability of PC-12 was decreased by H_2_O_2_ (Figure 7(d)), while the treatment with FA or NAC (a ROS scavenger) significantly recovered the viability of 200 *μ*M H_2_O_2_-induced PC-12 cells in a dose-dependent fashion (Figure 7(e)). Since the cell death induced by 6-OHDA or H_2_O_2_ was highly accompanied by the increase in intracellular ROS levels, we then investigated whether FA could decrease the production of ROS levels in 6-OHDA- or H_2_O_2_-treated PC-12 cells. As shown in the flow cytometric analysis in Figures 8(a) and 8(b), 6-OHDA and H_2_O_2_ significantly increased the ratio of cells with H_2_DCFDA fluorescence representing the ROS levels in cells, which was remarkably decreased by FA treatment. Together, our results demonstrate that FA can enhance the cell viability and reduce the ROS levels in 6-OHDA- or H_2_O_2_-treated PC-12 cells.

### 3.9. FA Inhibits Apoptosis in 6-OHDA- or H_2_O_2_-Treated PC-12 Cells

**Figure 9:**
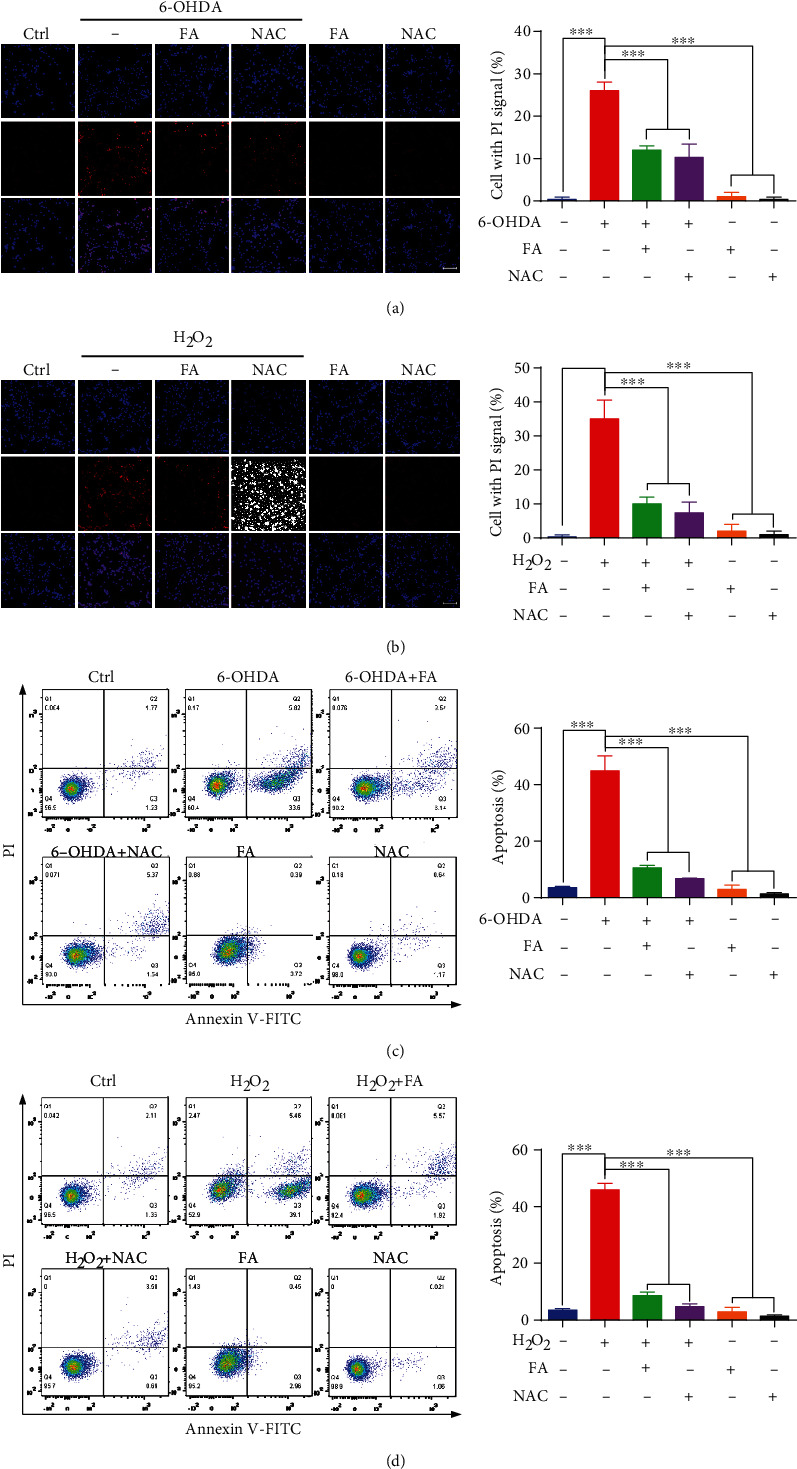
FA inhibits 6-OHDA- or H_2_O_2_-induced apoptosis in PC-12 cells. (a, b) PC-12 cells were treated with FA or NAC in the absence or presence of 6-OHDA or H_2_O_2_ at the indicated concentrations for 24 h. After treatment, cell apoptosis was determined by PI/Hoehcst33324 staining. Representative images of cells with Hoechst or PI signals were captured by using a fluorescence microscope (magnification: 20x). Scale bar: 100 *μ*m. The bar chart indicates the ratio of PI/Hoechst. Data were collected from three independent experiments and expressed as the mean ± SD. ^∗∗∗^*p* ≤ 0.001. (c, d) The cell viability was measured by flow cytometry using the Annexin V-FITC/PI apoptosis detection kit after the treatment with FA or NAC in the absence or presence of 6-OHDA or H_2_O_2_. The bar chart indicates the percentage of viability of PC-12 cells. Data were collected from three independent experiments and expressed as the mean ± SD. ^∗∗∗^*p* ≤ 0.001.

The emerging evidence indicates that 6-OHDA and H_2_O_2_ induce neuronal apoptosis, which is an important feature of PD. Thus, we investigated whether FA could protect neurons against 6-OHDA- and H_2_O_2_-induced apoptosis in PC-12 cells by the PI/Hochest33342 staining assay and flow cytometry analysis. As shown in Figures 9(a) and 9(b), PC-12 cells were observed with a high percentage of cells with the PI signal in cells with the Hoechst signal after exposure to 6-OHDA and H_2_O_2_ for 24 h, respectively, while pretreatment with FA significantly reduced the ratio of PI/Hoechst. In addition, 6-OHDA and H_2_O_2_ exposure significantly increased the apoptosis of PC-12 cells with the rate of 44.86% and 45.89%, respectively (Figures 9(c) and 9(d)). However, the pretreatment of FA could decrease the apoptosis rate to 10.54% and 8.64%. Together, these results suggest that FA can markedly protect the cell death of PC-12 cells by inhibiting apoptosis.

### 3.10. FA Protects PC-12 Cells Against 6-OHDA- or H_2_O_2_-Induced Injury via Autophagy Induction

**Figure 10:**
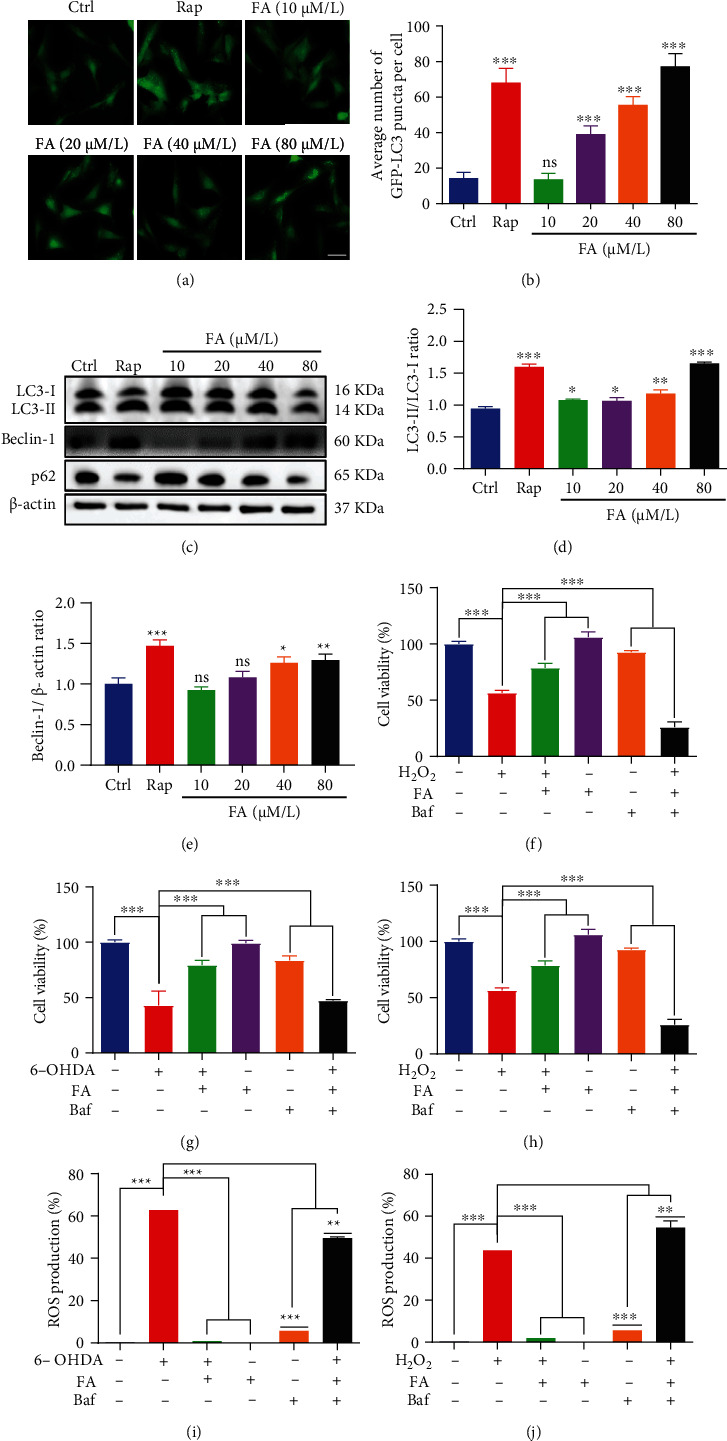
FA induces autophagy and inhibits 6-OHDA- or H_2_O_2_-induced cell death and ROS accumulation via autophagy activation in PC-12 cells. (a) Stable RFP-GFP-LC3 U87 cells were treated with FA at the indicated concentrations or Rap (0.5 *μ*M) for 24 h. Representative images with GFP-LC3 puncta formation were captured (magnification, ×40). Scale bar: 100 *μ*m. (b) The bar chart represents the number of GFP-LC3 puncta per cell. Bars, SD, ^ns^*p* > 0.05 and ^∗∗∗^*p* ≤ 0.001. (c–f) PC-12 cells were treated with FA or Rap at the indicated concentrations for 24 h. Then, cell lysates were harvested for the detection of LC3-I, LC3-II, Beclin 1, and SQSTM1/p62 proteins using Western blotting. The bar charts, respectively, indicate the ratio of LC3-II/LC3-I (d) and the levels of Beclin 1(e) and SQSTM1/p62 (f) proteins. Bars, SD, ^ns^*p* > 0.05, ^∗^*p* ≤ 0.05, ^∗∗^*p* ≤ 0.01, and ^∗∗∗^*p* ≤ 0.001. (g, h) 6-OHDA- or H_2_O_2_-induced PC-12 cells were treated with FA in the absence or presence of Baf for 24 h. After treatment, the cell viability was measured by the MTT assay. The bar chart indicates the viability of PC-12 cells. Data were collected from three independent experiments and expressed as the mean ± SD. ^∗∗∗^*p* ≤ 0.001. (i, j) 6-OHDA- or H_2_O_2_-induced PC-12 cells were treated with FA in the absence or presence of Baf for 24 h. After treatment, the cells were incubated with an H_2_DCFDA probe, and the GFP intensity was analyzed by flow cytometry. The bar charts present the GFP intensity indicating the ROS levels of PC-12 cells. Data were collected from three independent experiments and expressed as the mean ± SD. ^∗∗∗^*p* ≤ 0.001.

Increasing evidence indicates that autophagy plays a prominent role in maintaining cellular homeostasis via resisting cellular damage [31]. Therefore, we here investigated whether FA induced autophagy and improved 6-OHDA- and H_2_O_2_-induced injury via autophagy induction in PC-12 cells. As shown in Figures 10(a) and 10(b), FA treatment dose-dependently increased the formation of GFP-LC3 puncta in stable RFP-GFP-LC3 U87 cells. Additionally, the autophagic effect was also evaluated by measuring the conversion of LC3-I to LC3-II and the levels of Beclin 1 and SQSTM1/p62 proteins in PC-12 cells using the Western blotting method. The results showed that FA treatment significantly improved the ratio of LC3-II/LC3-I and Beclin 1 proteins and reduced the expression of SQSTM1/p62 proteins in a dose-dependent manner (Figures 10(c)–10(f)). Together, these data suggest that FA can induce autophagy in cells. Then, we employed Baf, an inhibitor of the late phase of autophagy, to inhibit the effect of FA on autophagy induction and determined the viability of 6-OHDA- and H_2_O_2_-treated PC-12 cells. The results showed that FA treatment significantly increased the viability of PC-12 cells in the presence of 6-OHDA or H_2_O_2_, while Baf successfully eliminated the protective effect of FA on rescuing 6-OHDA- or H_2_O_2_-induced cell death in PC-12 cells (Figures 10(g) and 10(h)). In addition, we also detected the ROS levels in 6-OHDA- or H_2_O_2_-induced PC-12 cells with the treatment of FA and Baf. The flow cytometry results showed that FA alone supplement remarkably reduced ROS levels in 6-OHDA- or H_2_O_2_-treated PC-12 cells, which were significantly eliminated by the addition of Baf (Figures 10(i) and 10(j)). Thus, these results suggest that FA improves cell viability and reduces ROS levels in 6-OHDA- or H_2_O_2_-induced PC-12 cells via activating autophagy.

## 4. Discussion

At present, as the population aging has risen rapidly worldwide, the incidence of neurodegenerative diseases has increased about 20-fold in the last 20 years, which brings heavy burdens on the healthcare system and society. PD is the second most common neurodegenerative disorder, usually occurring in people over 60 years old. Currently, there are still no drugs used for the cure of PD, and there is an urgent need to find novel and effective therapeutic approaches for PD.

Cumulative evidence has shown that the abnormal accumulation of *α*-synuclein in Lewy bodies and its induced progressive degeneration of DA neurons are the typical pathophysiological hallmarks of PD [32]. It has been demonstrated that *α*-synuclein aggregates can affect the functional integrity of neurons and induce neuronal toxicity, ultimately leading to neuronal death. Although the precise mechanisms related to *α*-synuclein have not been fully elucidated, converging evidence from genetic, pathological, and experimental studies has increasingly suggested that mitochondrial dysfunction, endoplasmic reticulum stress, lysosomal impairment, membrane disturbance, and synaptic dysfunction are strongly implicated in the *α*-synuclein pathology of PD [33]. These adverse physiological changes promote the massive production of ROS and eventually cause oxidative stress, which exacerbates the aggregation and spreading of *α*-synuclein, thus resulting in a vicious circle and further aggravating the symptoms of PD [33]. In addition, there is an amount of evidence indicating that PD neurotoxins such as 6-hydroxydopamine (6-OHDA), rotenone, and 1-methyl-4-phenyl-1,2,3,6-tetrahydropyridine (MPTP) have been widely used to induce PD models in vivo and in vitro [34]. They induce mitochondrial dysfunction, which increases ROS production and reduces cellular bioenergetics. Since neurons in the midbrain are metabolically very active with high mitochondrial energy demand and are especially vulnerable to oxidative stress, the exposure to these PD neurotoxins can cause mitochondrial impairment and ultimately neurotoxicity and the loss of nigrostriatal dopaminergic neurons [35]. Thus, the effective clearance of *α*-synuclein aggregates and the amelioration of neuronal damage induced by PD toxins have been suggested as attractive strategies for PD therapy. In this study, we employed *α*-synuclein, 6-OHDA, or H_2_O_2_ to establish PD models in vivo and in vitro.

Autophagy, commonly referred to as macroautophagy, is one of the major degradation pathways and plays a crucial role in maintaining the effective turnover of aggregated proteins and damaged organelles in cells [36]. Recently, the deficiency or blockade of autophagic flux leading to the accumulation of *α*-synuclein and/or damaged mitochondria has been observed in the brain of PD subjects [37], implying the key role of autophagy in PD. In turn, accumulating studies have demonstrated that the pharmacological enhancement of autophagy can alleviate neuronal death, the accumulation of *α*-synuclein, oxidative stress, and mitochondrial dysfunction [38]. For example, rapamycin is able to reduce *α*-synuclein accumulation and inhibit *α*-synuclein-induced neurodegeneration in wild-type, A30P, or A53T *α*-synuclein-expressing PC-12 cells [39] and in *α*-synuclein-overexpressing mice [40] and rats [41], respectively. In addition, lithium induces autophagy to prevent the accumulation of *α*-synuclein in PC-12 cells overexpressing A53T and A30P *α*-synuclein [42] and also protect against paraquat-induced neurotoxicity and cell death in neuronal cells [43]. Thus, targeting autophagy induction to discover the drugs will provide new opportunities for the treatment of PD.

In recent years, natural products have drawn great attention and have shown bright prospects in the discovery of novel autophagy enhancers and the treatment of neurodegenerative diseases such as PD. FA, a lignin-derived phenolic compound abundant in various natural sources, provides a broad spectrum of beneficial activities for human health. It has been widely tested for its pharmacological activities in vivo and in vitro, such as anti-inflammatory, anticancer, antiangiogenesis, antiapoptotic, and antioxidant properties [14]. However, the potential efficacy and underlying molecular mechanisms of FA in PD remain unclear. Here, by using multiple *C. elegans* models of PD, we found that FA significantly reduced the accumulation of *α*-synuclein and neurotoxicity induced by 6-OHDA in *C. elegans*. Meanwhile, FA also significantly improved the cell viability and inhibited the ROS levels and apoptosis in 6-OHDA- or H_2_O_2_-treated PC-12 cells. These results suggest that FA exerts beneficial effects on PD-related pathological processes, such as *α*-synuclein accumulation and oxidative damage. Further studies showed that FA could activate autophagy in both *C. elegans* and PC-12 cells. After inhibiting the autophagy activity of FA by feeding of RNAi bacteria, the effects of FA on the inhibition of *α*-synuclein and ROS accumulation and DA neurodegeneration in NL5901 or BZ555 worms were successfully abolished. Consistently, Baf also significantly attenuated the protective effect of FA on 6-OHDA- or H_2_O_2_-induced cell death and ROS production in PC-12 cells, suggesting that FA-induced autophagy can attenuate the oxidative damage of neurons [44]. However, further experiments are required to address the neuroprotective effect of FA on attenuating the cytotoxicity of *α*-synuclein, 6-OHDA, and H_2_O_2_ via autophagy induction in vitro and in vivo.

In conclusion, our current study demonstrated that FA improved the pathology of PD resulting from the accumulation of *α*-synuclein and the exposure of 6-OHDA and H_2_O_2_ via the activation of autophagy in *C. elegans* and PC-12 cells. These findings strongly suggest that FA may have considerable therapeutic applications for PD. In addition, further studies are essential to validate the anti-PD effect and explore the mechanism of action of FA.

## Data Availability

All the figures used to support the findings of this study are included within the article.
